# Severe mental illness and cardioprotective medication prescribing: a qualitative study in general practice

**DOI:** 10.3399/BJGPO.2023.0176

**Published:** 2024-06-26

**Authors:** Amanda Vettini, Gearóid K Brennan, Stewart W Mercer, Caroline A Jackson

**Affiliations:** 1 Centre for Population Health Sciences, Usher Institute, University of Edinburgh, Edinburgh, UK; 2 Faculty of Health Sciences & Sport, University of Stirling, Stirling, UK

**Keywords:** cardiovascular diseases, mental health, prescribing, qualitative research

## Abstract

**Background:**

Patients with severe mental illness (SMI) die 10–20 years earlier than the general population. They have a higher risk of cardiovascular disease (CVD) yet may experience lower cardioprotective medication prescribing.

**Aim:**

To understand the challenges experienced by GPs in prescribing cardioprotective medication to patients with SMI.

**Design & setting:**

A qualitative study with 15 GPs from 11 practices in two Scottish health boards, including practices servicing highly deprived areas (Deep End).

**Method:**

Semi-structured one-to-one interviews with fully qualified GPs with clinical experience of patients with SMI. Interviews were transcribed verbatim and analysed thematically.

**Results:**

Participants aimed to routinely prescribe cardioprotective medication to relevant patients with SMI but were hampered by various challenges. These structural and contextual barriers included the following: lack of funding for chronic disease management; insufficient consultation time; workforce shortages; IT infrastructure; and navigating boundaries with mental health services. Patient-related barriers included patients’ complex health and social needs, their understandable prioritisation of mental health needs or existing physical conditions, and presentation during crises. Professional barriers comprised GPs’ desire to practise holistic medicine rather than treating via cardioprotective prescribing in isolation, and concerns about patients’ medication concordance if patients were not prioritising this aspect of their health care at that particular time. In terms of enablers for cardioprotective prescribing, participants emphasised continuity of care as fundamental in engaging this patient group in effective cardiovascular health management. A cross-cutting theme was the current GP workforce crisis leading to ‘firefighting’ and diminishing capacity for primary prevention. This was particularly acute in Deep End practices, which have a high proportion of patients with complex needs and greater resource challenges.

**Conclusion:**

Although participants aspire to prescribe cardioprotective medication to patients with SMI, professional-, system- and patient-level barriers often make this challenging, particularly in deprived areas owing to patient complexity and the inverse care law.

## How this fits in

Patients with severe mental illness (SMI) have a marked excess risk of, and poorer outcomes from, cardiovascular disease (CVD). Quantitative research suggests that this patient group may experience suboptimal cardioprotective medication prescribing, but underlying reasons have not been investigated. Our study identified multiple challenges faced by GPs, with underfunding, workforce shortages, and insufficient consultation time affecting the continuity of care and strong doctor–patient relationships, identified as critical to providing optimal care for this complex-needs patient group. Urgent investment, and effective embedding of multidisciplinary teams in primary care, particularly in deprived areas, is needed to improve optimal cardioprotective medication prescribing in people with SMI.

## Introduction

Life expectancy of patients with an SMI, such as schizophrenia, bipolar disorder or major depression, is 10–20 years less than in the general population,^1–3^ which is largely owing to poorer physical health and in particular a higher burden of CVD. The association between SMI and increased risk of major cardiovascular disease events, such as heart attack and stroke, is well established.^4–6^ Pre-existing SMI is associated with lower short- and long-term survival following these events,^6–8^ as well as higher risks of recurrent vascular events.^9^ Suboptimal clinical care for CVD may play a role in these disparities,^6^ including undertreatment with guideline-recommended medication for cardiovascular risk factors or established disease.^7,9–11^ The reasons underlying the observed differences in cardiovascular medication by SMI status have been little investigated. Previous studies of GP experiences in managing patients with mental illness suggest that multiple factors influence or impede delivery of care more generally for this vulnerable group, including individual GP characteristics, confidence in treating patients with SMI, impediments to multidisciplinary care approaches, organisational issues, and patient-specific factors.^12,13^ The *Lancet Psychiatry Commission* blueprint for protecting physical health in patients with mental illness highlights the role of continuing care and improved use of medical investigations and treatments, including cardioprotective medications, in reducing physical disease burden in this group.^14^ Achieving this first requires better understanding of why patients with SMI are less likely to be prescribed cardioprotective medication when indicated. To our knowledge, no previous study has investigated GP experiences in this context. We therefore sought the experiences and concerns of, and challenges or barriers faced by, GPs when prescribing cardioprotective medication in patients with SMI. We purposively included ‘Deep End’ GPs working in deprived areas, given the concentration of patients with complex needs and mental–physical multimorbidity in such areas,^15^ and the limitations imposed on GPs by the inverse care law.^16^

## Method

This exploratory study took place in two health board areas in Scotland, with 10 participants from health board A and five from health board B. Participants were offered either a video or phone-call interview.

### Sampling and recruitment

We included participants who had completed GP training and had at least 1-year post-Certificate of Completion of Training (CCT) experience, were currently practising within the eligible health boards, and had regular contact with patients with SMI. We shared information about the study via multiple routes and existing clinical networks. This included publicising the study through the NRS Primary Care Network newsletter in Scotland, an email newsletter to eligible health board GPs, and contact with the ‘Deep End’ group.^17^ ‘Deep End’ refers to the GP practices serving the 100 most deprived populations in Scotland. These GP practices have ≥44% of their patients living at postcodes in the most deprived 15% of datazones, according to the Scottish Index of Multiple Deprivation (SIMD). GPs expressing an interest in participating were emailed an information leaflet and contacted at least 24 hours later to obtain informed consent and arrange an interview.

### Data collection and analysis

Data were collected via a pre-interview demographic questionnaire, to collect the following information: age group; sex; status in practice; contracted hours; year of GP qualification; time in current post; and qualifications in psychiatry or mental health. Data were also collected via an in-depth semi-structured qualitative interview of approximately 45 minutes. All interviews were conducted by video call except one that was conducted by telephone. Interviews were audio-recorded using an encrypted digital recorder, professionally transcribed verbatim, and anonymised assigning interviewee code numbers. The analytical process was data-driven and inductive; no codes were pre-assigned before fieldwork.

We used Braun and Clarke’s^18^ six-phase thematic analysis approach, which was used to guide our practice on the following: data familiarisation; coding; generating initial themes; developing and reviewing themes; defining, refining and naming themes; and formal writing-up. A first researcher (AV) conducted thematic analysis on the entire dataset using the full process outlined. A second researcher (GB) read all the interview transcripts and performed preliminary coding, and the two researchers discussed their views on coding and analysis, arriving at a shared analytical understanding. Provisional ‘candidate’ themes grouping codes with shared meaning were developed, reviewed for meaningful and accurate data storytelling, and named in analysis phases four and five. Mind and concept maps were generated in NVivo (version 12) to advance theoretical thinking and summarise key findings diagrammatically.

## Results

We recruited 15 GPs from 11 practices across the two health board areas. Ten GPs were from health board A and five from health board B; seven practices were from health board A and four from health board B. The sample comprised nine females and six males. Seven were GP partners, five were salaried, and three were locums or worked out of hours (OOH). Three participants identified themselves as having a postgraduate mental health qualification; the remainder did not view themselves as possessing a specialist postgraduate mental health qualification (Table 1).

**Table 1.** GP participant demographic data

Health board	Sex, *n*	Age band, *n*	GP role, *n*	Weekly contracted hours, *n*	Year qualified, *n*	Years at current practice, *n*	Postgraduate mental health qualification, *n*
Health board A, 10	Female, 9	35–39 years, 1	Partner, 7	≤20 hours, 8	1976–1999, 9	0–5 years, 2	Yes, 3
Health board B, 5	Male, 6	40–44 years, 2	Salaried(fixed term), 1	≥21 hours, 5	2000–2018, 6	6–10 years, 3	No, 12
		45–49 years, 2	Salaried(open ended), 4	N/A, 2		≥16 years, 7	
		50–54 years, 3	Locum or OOH, 3			N/A, 3	
		55–59 years, 3					
		60–64 years, 2					
		65–69 years, 0					
		≥70 years, 2					
**Totals**	**15**	**15**	**15**	**15**	**15**	**15**	**15**

OOH = out of hours.

GPs worked in a range of practices, although most were in deprived areas in line with the recruitment strategy. Seven practices were Deep End, one was relatively deprived (but not Deep End) and three were in affluent areas. Five of the practices were large (≥10 000 patients), two were medium sized (5000–9999), and four were small (0–4999). These totals sum to 11 and not 15 as two GPs worked at the same practice, and some were not affiliated to a particular practice, for example, locums and OOH GPs.

**Figure 1.**
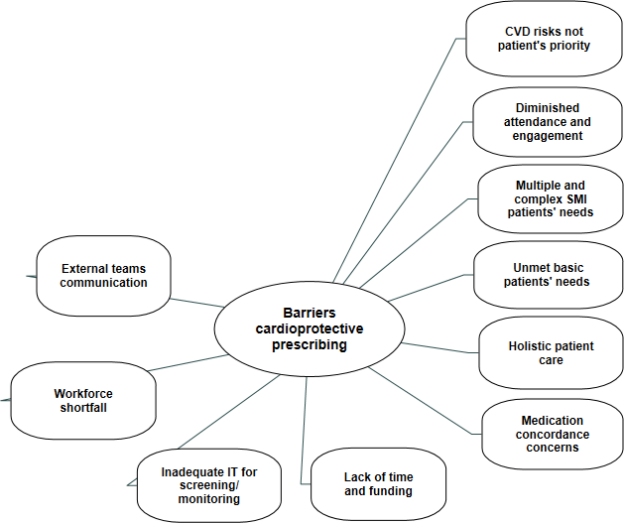
Factors identified by GP participants as being barriers to cardioprotective medication prescribing in people with severe mental illness. CVD = cardiovascular disease. SMI = severe mental illness

Findings below are structured around three key overarching themes: structural and contextual barriers; GP professional barriers and patient-related barriers (both Figure 1); and enablers to CVD medication prescribing. This section concludes by offering solutions proposed by interviewees regarding cardioprotective medication prescribing and chronic disease management in this population group. Each quote includes the participant number, stating whether or not they were working in a Deep End practice (denoted by DE or not DE). OOH is a GP who works out-of-hours or a locum. Edited participant quotes are shown in this paper, and the full version of each quote is provided in the supplementary file.

### Structural and contextual barriers

#### Workforce crisis in general practice

##### Lack of time and funding

Participants reported that the current general practice crisis reflects insufficient funding and workforce shortfall, owing to recruitment and retention issues. These contextual factors were felt to impact GPs’ capacity to provide optimum care, partly through further reducing available consultation time for proactive strategies around managing patients’ CVD risk and prescribing medication:

*‘The idea in a ten … minute appointment … while we’re talking about…* [being] *admitted to the* [local psychiatric hospital] *… I’m going to check your blood pressure and start you on a statin …’* (P6, DE)

##### Workforce shortfall and firefighting

Participants described the situation as ‘*firefighting*’, addressing immediate necessities rather than risk-factor horizon scanning. They indicated that primary prevention in this population group was hampered by the current infrastructure with demand outstripping supply and shrinking workforces impacting practices. They felt that recruitment and retention, particularly of GP partners and practice nurses, was impacting on chronic disease management and care for all patients:

*‘Two or three staff off sick … Two or three have resigned at any one point in time*.’ (P4, DE)

*‘We’re busy … essentially firefighting an awful lot in terms of medical conditions … that does affect our priorities … we’re probably less strategic … more just trying to get through the issues as they come up*.’ (P12, Not DE)

The consensus was that general practice was already in a suboptimal state when the COVID-19 pandemic started, owing to issues with the new 2018 GP contract and reduced funding. Participants felt that these issues had already impacted on care provision, including primary prevention, which has been exacerbated by the backlog of health problems that accumulated during lockdown. While participants indicated that practices are overall recovering the way they engage with patients, the workforce crises in general practice hindered their capacity to comprehensively deliver chronic disease management services.

### Team communication and waiting lists

Although participants praised mental health services, the long waiting list for referrals, exacerbated by the COVID-19 pandemic, created additional challenges for participants and their patients. Participants also noted the need for patients with SMI to consistently receive the mental health care they needed at the right time to make a difference. Where care from mental health teams was delayed, patients’ mental health was not stabilised. Participants reported this created urgency around mental health management, thus patients’ CVD risk could not be preventively treated, with neither patients nor GPs able to engage in this:

*‘Part of his* [mental] *illness means that he neglects himself ... So the main thing that I can do for him to help his physical health is to stabilise his mental health so that he is in the best position to be able to make informed choices*.’ (P5, DE)

Access to mental health teams and psychiatry services largely depended on how effective particular services were, as well as local working and communication practices. Provision varied greatly, reflecting a lack of a systematic infrastructure. For some GP practices, access to psychiatry services was fluid; for others, this was fraught with referral barriers and communication difficulties:

*‘Psychiatry services can be quite challenging … There are more barriers than ways of convening at … easy communication*.’ (P15, DE)

*‘It is quite difficult to really connect in with mental health services, at the moment … We refer patients, they’re often bounced back to us, we’re left trying to figure out what to do with them*.’ (P12, not DE)

### GP professional barriers

#### Holistic patient care

Participants viewed patients, their needs, and potential treatments holistically, seeing the ‘*whole person*’ rather than simply prescribing cardioprotective medication:

*‘The GP would take into account … physical … social, … psychological and make decisions based on a holistic view*.’ (P7, OOH)

Participants also reflected on whether lifestyle changes, such as smoking cessation or weight loss, would have larger impacts than prescribing, framed below as having ‘difficult’ conversations with patients:

*‘The medical bit is the tip of the iceberg. The easy bit is prescribing ... The hard bit is having the time to have all those really difficult but really important conversations*.’ (P5, DE)

#### Medication concordance concerns

Although most (11/15) of the GPs interviewed said they had previously initiated cardioprotective medication, they did not necessarily perceive this as straightforward, and did not support such prescribing for all patients with SMI and CVD risk factors. GP participants reported that they always continued prescribing cardioprotective mediation initiated elsewhere, such as in secondary care.Participants highlighted concerns about patients’ perceptions of the value of the prescribed medication and likely concordance. They were often reluctant to prescribe medication if non-concordance was perceived to be an issue:

*‘This medication is going to … long term … reduce your risk … patients that I deal with who have severe mental illness, are they going to understand that?’* (P3, Not DE)

Some participants reflected that they carefully considered whether a particular patient would tolerate additional medication(s) if already prescribed multiple medications. In a scenario where polypharmacy should be minimised, participants noted that they prioritise medications critical for acute health issues, and antipsychotics, for reducing emergency hospital admission.

### Patient-related barriers

#### Patient priorities

Participants frequently highlighted that, in their clinical experience, CVD features relatively low among the priorities of patients with SMI and is typically not their consultation motivation. They emphasised the need to address the patients’ priorities and the most pressing presenting issues (whether mental or physical) rather than instigating discussions around CVD risk and potential treatment. It was noted that patients with SMI frequently attend during mental health *‘crisis’*, which naturally takes treatment priority:

*‘I don’t think … it’s ever been the priority of the patient to address their cardiovascular health.’* (P2, DE)

*‘The highest priority is … making sure that they are safe from a mental health point of view … People present in crisis or with ongoing mental distress and so it doesn’t always feel appropriate to be talking about preventative medicine because it’s not their priority*.’ (P5, DE)

#### Multiple and complex needs, unmet basic needs, and engagement

Participants frequently highlighted the high level of socioeconomic deprivation in this group and concerns around lifestyle factors, given the high prevalence of smoking, excessive alcohol consumption, recreational drug misuse, physical inactivity, overweight and obesity, and poor diet.

Participants vocalised challenges for patients with SMI in accessing GP services owing to sometimes being unable to consistently attend appointments as their mental health impacted their capacity for forward planning:

*‘The biggest priority is more that they’re feeling down and can’t get out of bed*.’ (P2, DE)

### Enablers for cardioprotective prescribing

**Figure 2.**
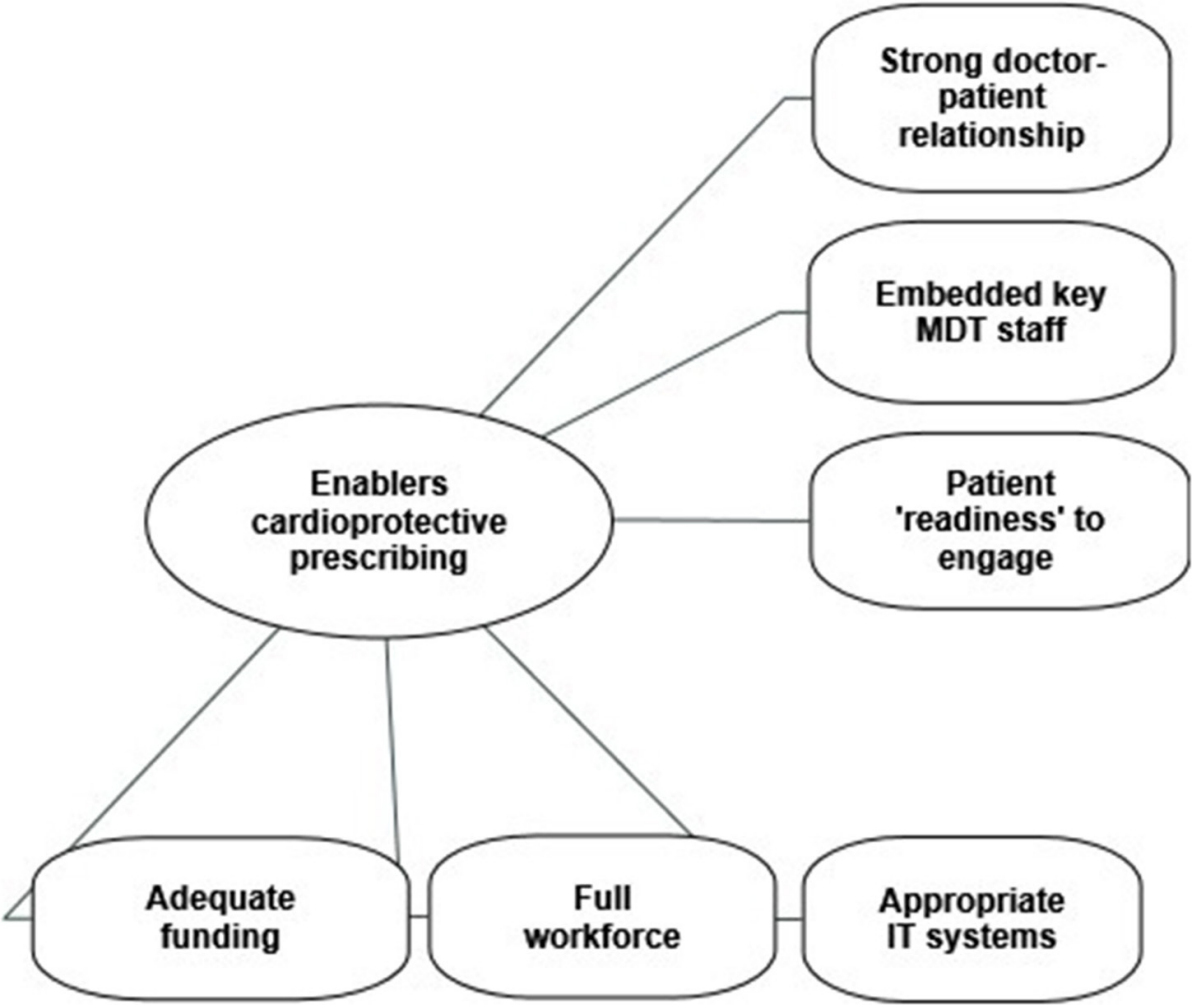
Factors identified by GP participants as being enablers to cardioprotective medication prescribing in people with severe mental illness. MDT = multidisciplinary team

Participants identified a number of factors that would help to enable cardioprotective medication prescribing and improve patient treatment engagement. Enablers included the following: relational continuity of care; a strong doctor–patient relationship; having key multi-disciplinary team (MDT) staff embedded in the practice; adequate funding; a full and stable practice workforce and appropriate IT systems; and patient readiness to engage (Figure 2), which was linked to two key factors: existing support networks; and having been diagnosed with a physical disease already.

#### Relational continuity of care and strong doctor–patient relationship

Participants often highlighted that the depth of the doctor–patient relationship and continuity of care within this population group, in particular, facilitates trust and improves receptiveness to their advice, which, in turn, improves likelihood of medication concordance and potential lifestyle change:

*‘Continuity is the thing that’s really important. A longer appointment is going to do ten times as much as ten short phone calls*.’ (P9, DE)

Some participants also noted that this relationship development was particularly important for patients with SMI experiencing homelessness, who frequently experience trust issues as a consequence of the stigma of homelessness. Doctor–patient relationships were highlighted as being especially crucial in socioeconomically deprived communities, with some participants noting the importance of GP workforce stability in fostering trust:

*‘We’re lucky, the patients know us. But … if you’ve got a massive turnover of GPs … you’re going to lose that doctor–trusted patient relationship. So you need to have GPs stable … Because mental illness, they’re terrified. They need to know who they’re talking to*.’ (P8, DE)

#### Patient engagement ‘readiness’

Some participants commented that patient readiness was important when initiating cardioprotective medication prescribing. Readiness was linked to two key factors, patient support networks and already being ‘in the system’. Participants linked sufficiently stable mental health and existence of a good informal support network of family and friends to creating a *‘mindset’* in a patient that is conducive to change:

*‘To … have an ongoing impact with change, folks need support … that often might be family members or friends*.’ (P12, Not DE)

Participants identified that patients already at high risk of further CVD were more likely to engage in their cardiovascular health and to be regularly monitored:

*‘If they’re on cardioprotective medication and … have a diagnosis of … hypertension or ischaemic heart disease … they will be on an annual recall … there’s more of a safety net for them*.’ (P5, DE)

By contrast, many participants commented that those not ‘in the system’ were most at risk of slipping through the net:

*‘Patients who’ve got potential physical health problems because of their severe mental illness. They’re the group that I think I worry about the most … not necessarily being screened … and are likely to be at risk of early death*.’ (P5, DE)

#### Functional IT systems for screening and monitoring

Some participants identified that their IT systems did not alert them that patients with SMI had not undergone their annual health check; others were concerned that such patients not already in the chronic disease monitoring system would be missed.

Participants frequently mentioned the new GP contract introduced in 2018, including changes associated with this such as ceasing the Quality Outcomes Framework (QOF) in Scotland in 2016. Although some GPs voiced criticisms of QOF, they noted its benefits in facilitating screening and monitoring of patients and efficient systems for identifying patients requiring follow-up, which has become more challenging without the support of QOF:

*‘QOF had lots of problems and became stupid and bloated but … switching off the digital tools to support the management of these patients, that was an unfathomably stupid decision*.’ (P6, DE)

Others emphasised the lack of IT systems’ interoperability between primary and secondary care.

### Aspirations and possible solutions

Interviewees offered several solutions to improving cardioprotective prescribing where indicated in patients with SMI. These solutions included the following: increasing the primary care workforce to full complement and sufficiently funding this workforce; improving IT screening and monitoring systems; enhancing communication across teams; providing integrated care; and consistently embedding much-needed MDT workers within practices (Table 2).

**Table 2.** Aspirations and solutions to facilitate cardioprotective prescribing

Bolstering and sufficiently funding primary care Improving IT systems for screening and monitoring Enhancing communication between physical and mental care teams Patients attending multidisciplinary team meetings Joint mental and physical health consultations Improving patients’ baseline quality of life through the provision of lifestyle community interventions Embedding core workers, for example, mental health nurse, pharmacist, link worker Providing key person for patient physical health discussions

#### Bolstering and funding the primary care workforce, embedding MDT workers

As outlined earlier, the shortfall in GP and other primary care workers severely impacts participants’ capacity to undertake primary prevention. Interviewees emphasised the need to increase the primary care workforce to improve delivery of care and enhance CVD prescribing conditions:

*‘If there was plenty of staff and plenty of time … you might be able to inch the uptake and prescribing rates*.’ (P14, OOH)

Although the 2018 GP contract aimed to embed MDT staff in primary care, participants highlighted the lack of consistency with which this has occurred. Several participants noted the critical importance of embedding core professionals, such as mental health nurses, pharmacists, and link workers (referred to as the ‘*Holy Trinity*’), within primary care. Interestingly, one GP in the quote below commented that this ‘*Holy Trinity*’ should include a financial support worker. Participants commented on difficulties contacting external pharmacists and mental health services. This was not an issue where such services were embedded within the practice, as patients did not need to be referred externally. Participants without embedded MDT workers aspired to this team structure:

*‘Holy Trinity … that would make a massive difference … Three people, your mental health worker, your link worker and your financial support advisor*.’ (P8, DE)

Some participants also proposed that each patient with a SMI should be designated a single primary care worker (not necessarily a GP), responsible for engaging in detailed discussions about patients’ physical and mental health and optimal responses, appropriate to their professional role.

#### Integrated care, enhanced communication

Participants identified solutions regarding improved integrated care and better communication between different care teams, and joint physical and mental health team consultations that potentially included patients:

*‘Joint consultations … with a psychiatrist … a dietitian … CPNs* [community psychiatric nurses]*, and the patient*.’ (P7, OOH)

A GP commented on a more strategic view of how chronic disease may be addressed in the future, advocating that this should be led by Community Health Care Partnerships (CHCPs) and with integrated, co-located mental and physical care.

#### Lifestyle community interventions

CVD risk management via prescription medication is only one potential avenue; many participants argued that social prescribing and lifestyle change remained the greatest solution to reducing cardiovascular risk in patients with SMI:

*‘20-minute neighbourhoods, free public transport, free Edinburgh Leisure, bicycles. If you’re wanting to make a big difference, that’s the level where you’re going to make population changes*.’ (P9, DE)

## Discussion

### Summary

The GPs interviewed in our study consider general practice in Scotland to be in crisis. Underfunding, workforce shortages, and insufficient consultation time reduce possibilities for the continuity of care and strong doctor–patient relationships, which are essential to provide optimal cardiovascular care in people with SMI. Consequently, ‘*firefighting*’ practices become necessary and primary prevention unavoidably affected. Participants perceived that CVD risk was of less importance to patients with SMI, given their highly complex health and social care needs. Prescribing cardioprotective medication was viewed as sometimes being at odds with practising holistic medicine. Participants were concerned about cardioprotective medication concordance owing to the multiplicity of complex healthcare and social needs in these patients’ lives. Many participants did aspire to more routinely initiating cardioprotective medication for this patient group and proposed solutions.

### Strength and limitations

Our study has a number of strengths. We achieved good representation from both geographical areas and in terms of sex, job status, and practice deprivation level. Fifteen interviews is a reasonable sample size for qualitative research, especially for short exploratory studies.^19^ Interview data showed a high degree of similarity, indicating that data saturation (that is, no new data emerging) was likely reached. To the best of our knowledge, our study is the first to explore GPs’ views on cardioprotective prescribing for this patient group. It provides important insight, especially in helping to understand findings from quantitative studies on this topic.

Our study is limited in that we included participants from the Scottish central belt region and so included largely urban practice participants. Thus, we could not explore issues relevant to remote and rural practices. Our study sought to include GPs only, but additional insight could be gained from interviewing other healthcare professionals and patients.

### Comparison with existing literature

Participants’ views on diminished cardioprotective prescribing and the primary prevention ‘safety net’ that having an existing physical condition provides, resonates with a study that French GPs are more likely to prescribe statins to general population patients who have diabetes and multimorbidity than to patients without diabetes.^20^ The views of participants in our study around inconsistent appointment attendance by patients with SMI and reduced engagement with some of their physical health care contrasts somewhat with findings from other studies. Recent Australian studies involving patient participants found they prioritise their physical health, but experience barriers and want support with this prioritisation.^21,22^ The evidence is mixed regarding GP attendance by patients with SMI. Our findings align with those of other studies, which indicate how difficult it is for patients with SMI to keep commitments owing to mental distress, medication effects, insomnia, and other problems.^23^ However, this contrasts with other study findings. For example, two quantitative studies reported increased consultation rates among patients with SMI versus without SMI in the UK.^10,24^ However, these studies used data entirely or largely from England, where QOF operates within general practice. The discontinuation of QOF in Scotland may partly account for the views of participants in our study. Our study participants’ views of the 2018 GP contract align with those from a recent Scottish primary care study that found sluggish progress on MDT expansion, lack of substantial reduction of participants’ workload, enduring time deficits, and lack of care enhancement for patients with complex needs.^25^ Their reflections on continuing workforce problems in general practice also resonate with a commentary on the UK GP workforce recruitment and retention crisis.^26^

Voorhees *et al*’s work around patients’ health service access^27^ cautions against a reductionist approach focusing on appointment promptness and number. Drawing on Levesque *et al* ^28^ they define access as the ‘*human fit*’ between patients’ ‘*needs and abilities*’ to access and healthcare professionals’ ‘*capacity and abilities*’ to provide services. Considering this definition in relation to our findings, the general practice workforce crisis and lack of funding compromise GPs’ capacity and ability to prevent CVD. Moreover, patients’ ability to ‘perceive’ their CVD needs is affected by their mental health being their central preoccupation and their difficulties managing daily life. Consequently, they can experience great challenges in seeking, reaching, and engaging^27^ with healthcare professionals. As argued by other studies on associations between early onset multimorbidity, deprivation, and mental health disorders,^29^ true continuity of care and the right conditions to foster depth of the doctor–patient relationship need to be foregrounded, as opposed to focusing on timely and numerous appointment provision (as policy targets have typically emphasised).

### Implications for research and practice

While our study provides valuable initial insight into the challenges faced by GPs when prescribing cardioprotective medication to people with SMI, further research is needed to determine whether these findings can be extrapolated to GPs across Scotland and the rest of the UK. Our findings warrant investment in a larger mixed-method study that includes both GPs and patients with SMI and incorporates survey and interview approaches, as well as potentially observation (either through video recording or direct observation) of GPs’ consultations with this patient group. While GPs in our study indicated diminished engagement in physical health issues among this patient group, evidence indicates that this does not reflect a lack of desire to improve their physical health. Motivational interviewing^30^ could be a useful strategy if GPs encounter difficulties when advising positive behavioural change.

Our study provides further evidence supporting the need for strategic investment in primary care infrastructure in Scotland. Patients with mental illness and physical comorbidity represent the tip of the iceberg of patients with multimorbidity and complex needs, with our study highlighting key challenges that likely impact other vulnerable patient groups. Key implications include the need to urgently address workforce shortfalls and consistently embed effective multidisciplinary teams in general practice, both of which would, in turn, facilitate more consistent annual physical health checks for this patient group. Cardioprotective medication is one element of holistic cardiometabolic care for this patient group, alongside lifestyle modification advice and antipsychotic medication optimisation, which collectively support improving overall physical health. Our findings also highlight the need for strategic use of existing funding and a change in policy focus, to allow prioritisation of improved continuity of care. Such improvement is critical to facilitating opportunities for patients with SMI to build the trusted doctor–patient relationship. This relationship is required to increase receptivity to preventive treatments and improve the potential for upstream and proactive care, as opposed to the firefighting and downstream medicine practices that appear to be currently necessary.

## Data

The dataset relied on in this article may be available from the corresponding author on reasonable request.
